# Crystal structure of tRNA m^1^A58 methyltransferase TrmI from *Aquifex aeolicus* in complex with *S*-adenosyl-l-methionine

**DOI:** 10.1007/s10969-014-9183-0

**Published:** 2014-06-04

**Authors:** Mitsuo Kuratani, Tatsuo Yanagisawa, Ryohei Ishii, Michiyo Matsuno, Shu-Yi Si, Kazushige Katsura, Ryoko Ushikoshi-Nakayama, Rie Shibata, Mikako Shirouzu, Yoshitaka Bessho, Shigeyuki Yokoyama

**Affiliations:** 1 RIKEN Genomic Sciences Center, 1-7-22 Suehiro-cho, Tsurumi-ku, Yokohama, 230-0045 Japan; 2 RIKEN Structural Biology Laboratory, 1-7-22 Suehiro-cho, Tsurumi-ku, Yokohama, 230-0045 Japan; 3 Department of Biophysics and Biochemistry, Graduate School of Science, The University of Tokyo, 2-11-16 Yayoi, Bunkyo-ku, Tokyo 113-0032 Japan; 4 Division of Structural and Synthetic Biology, RIKEN Center for Life Science Technologies, 1-7-22 Suehiro-cho, Tsurumi-ku, Yokohama, 230-0045 Japan; 5 RIKEN SPring-8 Center, 1-1-1 Kouto, Sayo, Hyogo 679-5148 Japan

**Keywords:** AdoMet, tRNA modification enzyme, Methylation, X-ray crystal structure, Structural genomics

## Abstract

The *N* ^1^-methyladenosine residue at position 58 of tRNA is found in the three domains of life, and contributes to the stability of the three-dimensional L-shaped tRNA structure. In thermophilic bacteria, this modification is important for thermal adaptation, and is catalyzed by the tRNA m^1^A58 methyltransferase TrmI, using *S*-adenosyl-l-methionine (AdoMet) as the methyl donor. We present the 2.2 Å crystal structure of TrmI from the extremely thermophilic bacterium *Aquifex aeolicus*, in complex with AdoMet. There are four molecules per asymmetric unit, and they form a tetramer. Based on a comparison of the AdoMet binding mode of *A. aeolicus* TrmI to those of the *Thermus thermophilus* and *Pyrococcus abyssi* TrmIs, we discuss their similarities and differences. Although the binding modes to the N6 amino group of the adenine moiety of AdoMet are similar, using the side chains of acidic residues as well as hydrogen bonds, the positions of the amino acid residues involved in binding are diverse among the TrmIs from *A. aeolicus*, *T. thermophilus*, and *P. abyssi*.

## Introduction

Posttranscriptional modifications alter the characteristics of tRNAs in various manners, to fine-tune their functions. The modified nucleoside *N* ^1^-methyladenosine is found at four positions: position 9 of mammalian mitochondrial tRNAs, position 14 of mammalian cytoplasmic tRNA^Phe^, position 22 of tRNA in some bacteria, and position 58 of tRNA in the three domains of life [1]. The *N* ^1^-methylation of adenosine abrogates its ability to form a standard Watson–Crick base pair, as also found with m^1^G, m^1^I, m^3^C, m^3^U, and m^3^Ψ. Indeed, reverse transcriptases read m^1^A with very low efficiency, and those in the HIV-1 and Molony murine leukemia viruses utilize the host’s tRNA bearing m^1^A for their replication [2–5].

In the absence of the m^1^A9 modification, mammalian mitochondrial tRNA^Lys^ could adopt an extended hairpin structure that is unproductive in translation, since an undesired base pair between A9 and U64 is tolerated [6, 7]. In yeast, the strain with a defective m^1^A58 modification is nonviable, because the initiator tRNA^Met^ is degraded [8]. In the native yeast tRNA, the m^1^A58 of the initiator tRNA^Met^ forms the reverse Hoogsteen base pair with A54, which increases the stability of the three-dimensional structure, while the m^1^A58 in the other 19 tRNAs forms the reverse Hoogsteen base pair with T54 [9–11]. In the thermophilic bacterium *Thermus thermophilus*, inactivation of the *trmI* gene results in a thermosensitive phenotype, suggesting that the m^1^A58 modification is important for both thermal adaptation and tRNA stability [12]. The m^1^A58 residue was analyzed by NMR and IR spectral studies, which considered the ^1^H, ^13^C, and ^15^N chemical shifts, the consistency of the sugar pucker and glycosidic conformations with those of the X-ray structure, and the character of the bond between the C6 and N6 atoms [13, 14]. Based on the results, the m^1^A58 residue in the native tRNA was deduced to be fully protonated, with its charge probably dislocalized from the quaternary N1 atom toward the C6, C5, and C4 atoms. The protonated state of the m^1^A58 residue is characteristic of the Mg^2+^-bound native state, and the partial charge in the tRNA elbow region may affect its interaction with the translational machinery [13, 14]. Therefore, the m^1^A58 modification of tRNA is important for stabilizing the L-shaped structure and for efficient translation [15].

The methyl group of m^1^A58 is transferred from the methyl donor *S*-adenosyl-l-methionine (AdoMet) by the TrmI homotetramer in bacteria and archaea, and by the Trm6/Trm61 α2/β2 heterotetramer complex in eukaryotes [8, 12]. The coordinated structural genomics projects on proteins from *Mycobacterium tuberculosis* determined the first structure of TrmI, as the conserved hypothetical methyltransferase Rv2118c [16]. At the same time, an in silico fold prediction study was reported [17]. Subsequently, the crystal structure of the catalytic domain (residues 70–250) of the TrmI tetramer from *Pyrococcus abyssi* revealed its mechanism of thermal stabilization, using intersubunit disulfide bonds [18]. The crystal structure of TrmI from *T. thermophilus* [19] was determined and complemented by biophysical characterizations, which revealed the tRNA binding stoichiometry per TrmI tetramer [19]. The crystal structure of full-length TrmI from *P. abyssi* was reported with further biochemical characterization of the region specificities [20]. Presently, eight PDB datasets from six species are available, and their structural architectures have been compared [21]. Comprehensive structural genomics projects on a specific organism, typified by that on *M. tuberculosis* [22], have provided the structural basis to characterize the biological functions of the proteome, including conserved proteins with unknown functions. On the other hand, comparative analyses of large numbers of orthologous and homologous structures, including some acquired by high-throughput capability and successful structural genomics [23–25], will lead to further understanding of the structure–function relationships of proteins and facilitate applications, including protein engineering and drug design. Here, we report the crystal structure of TrmI from *Aquifex aeolicus* in the complex with AdoMet, determined at 2.2 Å resolution. The overall tetrameric architecture is quite similar to the structures of TrmIs from other species [21]. We examined the similarities and differences in the AdoMet recognition by *A. aeolicus* TrmI, as compared to those by the TrmIs from *T. thermophilus* and *P. abyssi*.

## Materials and methods

### Cloning, expression, and purification of *A. aeolicus* TrmI

The aq_311 gene, encoding the *A. aeolicus* TrmI protein (gi: 15605836) comprising 248 residues, was amplified by PCR using *A. aeolicus* VF5 genomic DNA and cloned into the pET-21a expression vector (Merck Novagen, Darmstadt, Germany). The expression vector was transformed into the *E. coli* Rosetta™ (DE3) strain (Merck Novagen). The cells were cultured at 37 °C in LB medium, supplemented with 30 µg/ml chloramphenicol and 50 µg/ml ampicillin. The protein expression was induced by 0.5 mM IPTG. Following an overnight incubation, the cells were harvested by centrifugation and stored at −80 °C. The cells were resuspended in 20 mM Tris–HCl buffer (pH 8.0), containing 300 mM NaCl, 5 mM MgCl_2_, 0.5 mM EDTA, and 1 mM DTT, and were lysed by sonication on ice. The cell lysate was heat-treated at 70 °C for 30 min to denature most of the *E. coli* proteins, and was centrifuged at 15,000×*g* for 20 min at 4 °C. The supernatant was desalted by dialysis against 20 mM Tris–HCl buffer (pH 8.0) containing 1 mM DTT, and applied to a HiTrap Q column (GE Healthcare Biosciences), equilibrated with the same buffer. The protein was eluted with a linear gradient (0–1.0 M) of NaCl, and the target fractions, which eluted around 0.4 M NaCl, were collected. Ammonium sulfate was added to the sample, which was applied to a Resource PHE column (GE Healthcare Biosciences), equilibrated with 20 mM Tris–HCl buffer (pH 8.0) containing 1.2 M ammonium sulfate and 1 mM DTT, and was eluted with a decreasing linear (1.2–0 M) gradient of ammonium sulfate. The target fractions, which were eluted in 0.6–0.3 M ammonium sulfate, were collected and desalted by dialysis. The sample was applied to a Mono S column (GE Healthcare Biosciences), equilibrated with 20 mM Tris–HCl buffer (pH 8.0) containing 1 mM DTT, and was eluted by a linear (0–1.0 M) gradient of NaCl. The fraction that eluted at 0.3 M was concentrated and applied to a HiLoad 16/60 Superdex 75 pg column (GE Healthcare Biosciences), equilibrated with 20 mM Tris–HCl buffer (pH 8.0) containing 150 mM NaCl and 1 mM DTT. The gel filtration elution profile showed one peak at 50 ml, which corresponds to 0.41 column volumes. The protein sample was concentrated to 15 mg/ml by ultrafiltration. The protein purification was analyzed by SDS-PAGE. The electrophoretic mobility of *A. aeolicus* TrmI is almost the same as that of a marker (29 kDa), in agreement with its theoretical molecular weight (28.7 kDa). The final yield was 2.2 mg/l of culture.

### Crystallization and data collection

The *A. aeolicus* TrmI protein at 10–12 mg/ml concentrations, in 20 mM Tris–HCl buffer (pH 8.0) containing 150 mM NaCl, 1 mM DTT, and 2 mM AdoMet, was used for crystallization. Initial crystallization screening was performed in 1:1 sitting-drop vapor-diffusion reactions at 20 °C, by mixing 1 μl protein solution with 1 μl reservoir solution. The crystals were grown in 0.1 M Tris–HCl buffer (pH 8.4) and 20 % ethanol. The crystals were transferred to 0.1 M Tris–HCl buffer (pH 8.4), 20 % ethanol, and 35 % ethylene glycol for cryoprotection, prior to flash-cooling in liquid nitrogen for data collection. The native dataset was collected on beamline BL41XU at SPring-8 (Table 1). Data collected from a single crystal at 100 K were processed with the *HKL2000* program [26].

**Table 1:** X-ray data and refinement statistics

	*A. aeolicus* TrmI
Crystal parameters	
Space group	*P* 2_1_ 2_1_ 2_1_
Cell dimensions	
a, b, c (Å)	69.8, 97.2, 212.7
α, β, γ (°)	90, 90, 90
Matthews coefficient (Å^3^/Da)	3.14
Solvent content (%)	60.9
Data collection	
Wavelength (Å)	1.00
Resolution (Å)	50–2.2 (2.28–2.2)
*R*_sym_ (%)^a^	3.3 (43.9)
No. of unique reflections	68,373
No. of reflections in *R*_free_ set	3,597
Mean redundancy	6.6 (3.6)
Overall completeness (%)	96.7 (77.0)
Mean I/σ	23.7 (4.1)
Refinement residuals	
Resolution (Å)	50–2.2 (2.26–2.2)
*R*_free_ (%)^b^	23.0 (26.3)
*R*_work_ (%)^b^	19.4 (20.7)
Completeness (%)	96.8 (75.3)
Model quality	
RMSD bond lengths (Å)	0.008
RMSD bond angles (°)	1.1
Molprobity Ramachandran distribution	
Most favored (%)	98.6
Allowed (%)	1.4
Disallowed (%)	0.0
Mean main chain B-factor (Å^2^)	26.5
Mean overall B-factor (Å^2^)	31.7
Mean ligand B-factor (Å^2^)	32.3
Mean solvent B-factor (Å^2^)	31.2
Model contents	
Protomers in ASU	4
Protein residues	2–248
Ligands	4 AdoMet
No. of protein atoms	8,092
No. of ligand atoms	108
No. of water molecules	537
PDB accession code	2YVL

*RMSD* root-mean-square-deviation, *ASU* asymmetric unit

^a^ *R*_sym_ = Σ_*hkl*_ Σ_*j*_|*I*_*j*_(*hkl*) − <*I*_*j*_(*hkl*)>|/Σ_*hkl*_Σ_*j*_ *I*(*hkl*), where *I*_*j*_(*hkl*) and <*I*_*j*_(*hkl*)> are the intensity of measurement *j* and the mean intensity for the reflection with indices *hkl*, respectively

^b^ *R*_work, free_ = Σ|*F*_obs_ − *kF*_calc_|/Σ_*hkl*_ *F*_obs_, where *k* is a scale factor, and the crystallographic *R*-factor is calculated including (*R*_work_) and excluding (*R*_free_) reflections. In each refinement, free reflections consist of 5 % of the total reflections

### Structure solution and refinement

The phase was determined by the molecular replacement method, using the coordinates of TrmI from *Thermotoga maritima* (PDB ID: 1O54) as the starting model, with the program MOLREP [27]. The model was completed using iterative cycles of manual rebuilding in Coot [28] and computational refinement at 2.2 Å in *Refmac5* [29] (Table 1).

### Structure validation and deposition

The structure validation of the model is summarized in Table 1. The atomic coordinates and structure factors have been deposited in the Protein Data Bank, under the accession code 2YVL.

### Sedimentation velocity ultracentrifugation analysis

The *A. aeolicus* TrmI protein, at a 1 mg/ml concentration in 20 mM Tris–HCl buffer (pH 8.0) containing 150 mM NaCl and 1 mM DTT, was analyzed by ultracentrifugation at 20 °C, in a ProteomeLab XL-I ultracentrifuge (Beckman Coulter) with the An-60 Ti analytical rotor. The sample was ultracentrifuged at 40,000 rpm, and the absorbance at 280 nm was measured. The data were analyzed and the distribution c(M) was calculated by Sedfit [30].

## Results and discussion

**Fig. 1:**
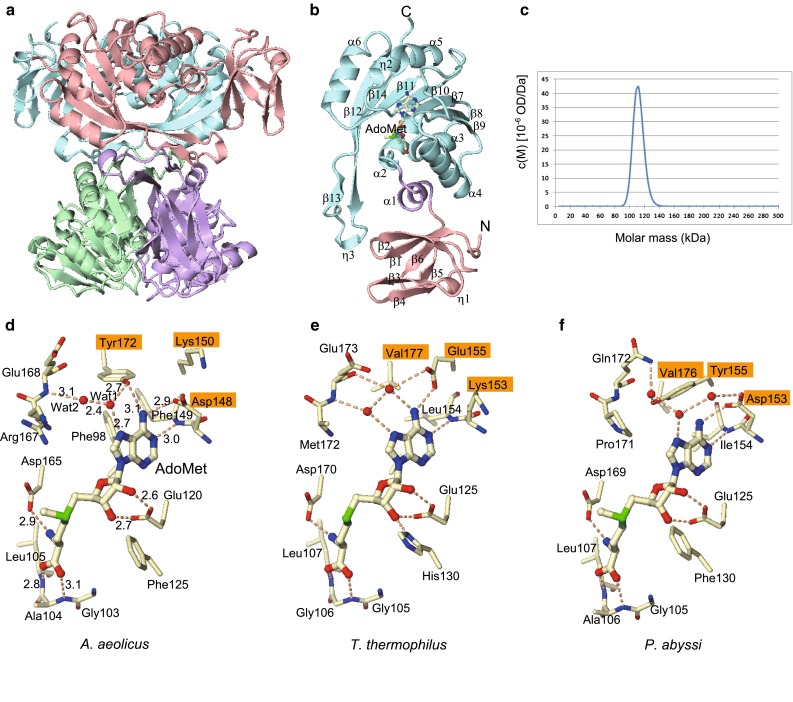
Crystal structure of *A. aeolicus* TrmI in complex with AdoMet. **a** The tetrameric structure of *A. aeolicus* TrmI. The four protomers are colored *pink*, *cyan*, *purple*, and *green*. **b** Protomer structure of *A. aeolicus* TrmI. The N-terminal domain, the linker helix, and the C-terminal domain are colored *pink*, *purple*, and *cyan*, respectively. The secondary structures are labeled. **c** The calculated distributions c(M) by Sedfit [30]. **d**–**f** *Ball-and-stick* representations of AdoMet binding by *A. aeolicus* TrmI (chain A) (**d**), *T. thermophilus* TrmI [19] (chain A) (**e**), and *P. abyssi* TrmI [20] (chain A) (**f**). The three amino acid residues surrounding the N6 amino group of AdoMet are labeled with *orange rectangles*. Hydrogen bonds are depicted by *dotted lines* with their distances (Å). The figures were created using CueMol (http://cuemol.sourceforge.jp/en/)

The crystal structure of *A. aeolicus* TrmI was determined at 2.2 Å resolution by the molecular replacement method, and was refined to *R*_work_ and *R*_free_ factors of 19.6 and 23.0 %, respectively (Table 1). The asymmetric unit contains four protomers (A–D) (Fig. 1a) and four AdoMet molecules. The electron density was interpretable for 247 residues (Asn2–Thr248). The *A. aeolicus* TrmI protomer (Fig. 1b) consists of the small N-terminal domain (residues 2–58) and the C-terminal methyltransferase domain (residues 72–248), which are connected by an α-helical linker (residues 59–71). The N-terminal domain forms a small β sandwich (Fig. 1b), in which the β sheet β2–β1–β6–β5 stacks on the β hairpin β3–β4, along with the small 3_10_-helix η1. The C-terminal domain adopts the typical type I methyltransferase fold, with a central seven-stranded β sheet with the topology β9–β8–β7–β10–β11–β14–β12, flanked by α helices on both sides (Fig. 1b). As reported previously [19], the long β strand β12, in which the head interacts with β13, is characteristic of TrmI among the type I methyltransferases, and it provides a surface for tetramerization.

We analyzed the oligomeric state of *A. aeolicus* TrmI in solution by sedimentation velocity ultracentrifugation. The gel filtration elution profile of *A. aeolicus* TrmI showed one peak between the IgG (158 kDa) and human albumin (66 kDa) markers. Since the theoretical molecular weight of *A. aeolicus* TrmI is 28.7 kDa, TrmI is suggested to exist as tetramer (114.8 kDa) in solution. The ultracentrifugation analysis, using 1 mg/ml *A. aeolicus* TrmI, showed one peak at 110 kDa (Fig. 1c), which confirmed that it is tetrameric in solution.

The methyl donor AdoMet is bound in the C-terminal domain of the protein (Fig. 1b). *A. aeolicus* TrmI recognizes AdoMet by hydrogen bonds from its main-chain and side-chain atoms as well as water-mediated hydrogen bonds (Fig. 1d), in a similar manner to *T. thermophilus* TrmI (Fig. 1e) [19] and *P. abyssi* TrmI (Fig. 1f) [20]. The N1 atom of the adenine moiety hydrogen bonds with the main chain amide nitrogen of Phe149 (3.0 Å) of *A. aeolicus* TrmI. The N6 amino group hydrogen bonds with the side chains of Asp148 (2.9 Å) and Tyr172 (3.1 Å) (Fig. 1d). The N7 atom interacts with a water (wat1 in Fig. 1d; 2.7 Å), which participates in a hydrogen bonding network involving Glu168, Tyr172, and a water (Wat2 in Fig. 1d). In addition to these four hydrogen bonds, the adenine ring forms a T-stacking interaction with the side chain of Phe98, which is fixed by π–π stacking with that of Phe149 (Fig. 1d). The two hydroxyl groups of the ribose moiety of AdoMet interact with the side chain of Glu120 (2.6 and 2.7 Å; Fig. 1d). The methionine moiety of AdoMet forms three hydrogen bonds (Fig. 1d): its amino group hydrogen bonds with the side chain of Asp165 (2.9 Å), and its carboxyl group hydrogen bonds with the main-chain amide nitrogen atoms of Ala104 (3.1 Å) and Leu105 (2.8 Å; Fig. 1d).

**Fig. 2:**
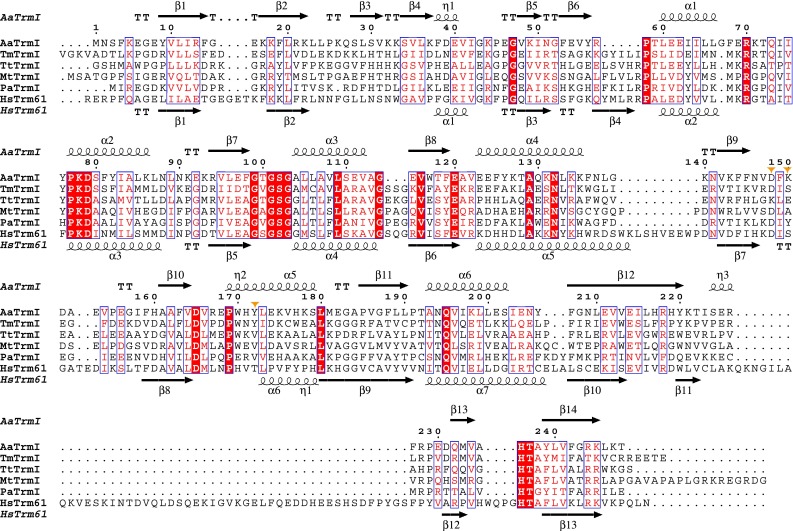
Sequence alignment of TrmI proteins. The amino acid sequences of *A. aeolicus* TrmI (AaTrmI), *T. maritima* TrmI (TmTrmI), *T. thermophilus* TrmI (TtTrmI), *M. tuberculosis* TrmI (MtTrmI), *P. abyssi* TrmI (PaTrmI), and *H. sapiens* Trm61 (HsTrm61) were aligned with ClustalX 2.1 [31]. Identical residues are *white in a red background*. Similar residues are *red in blue rectangles*. The secondary structures of *A. aeolicus* TrmI (PDB: 2YVL) and *H. sapiens* Trm61 (PDB: 2B25) are shown at the *top* and *bottom*, respectively. The three amino acid residues with side chains located near the N6 amino group of AdoMet are indicated by *orange triangles*. The figure was depicted by ESPript [32]

We compared the structure of *A. aeolicus* TrmI to those of *T. thermophilus* TrmI in complex with *S*-adenosyl-l-homocysteine (AdoHcy) (Fig. 1e) and *P. abyssi* TrmI in complex with AdoMet (Fig. 1f), and examined the conservation of residues involved in AdoMet binding. *A. aeolicus*, *T. thermophilus*, and *P. abyssi* all live in high-temperature environments. The N6 amino group of the adenine moiety is recognized in diverse manners by the various TrmI structures. The side chains of three amino acid residues (Asp148, Lys150, and Tyr172 in *A. aeolicus* TrmI; Fig. 2) surround the N6 amino group, and the underlined residues are involved in AdoMet binding. In the corresponding three positions, *T. thermophilus* TrmI has Lys153, Glu155, and Val177, while *P. abyssi* TrmI has Asp153, Tyr155, and Val176 (Fig. 2). Asp148 and Tyr172 of *A. aeolicus* TrmI form direct hydrogen bonds with the N6 amino group (Fig. 1d). In *T. thermophilus* TrmI (Fig. 1e) [19], the side chain of Glu155 and a water molecule form hydrogen bonds with the N6 amino group, and these are apparently equivalent to the two hydrogen bonds formed between this moiety and *A. aeolicus* TrmI. However, Glu155 of *T. thermophilus* TrmI is located at a different position than Asp148 of *A. aeolicus* TrmI in the amino acid alignment (Fig. 2). On the other hand, *P. abyssi* TrmI forms only one hydrogen bond by Asp153 (Fig. 1f) [20], which is located at the same position as Asp148 of *A. aeolicus* TrmI (Fig. 2). The distances from the N6 amino group to the three water molecules (Fig. 1f) are 3.8, 3.9, and 5.5 Å, respectively. The N7 atom of AdoMet is bound to TrmI by one water-mediated hydrogen bond, although the side chains involved in its coordination differ (Fig. 1d–f).

We examined the conservation of these three amino acid residues in the other TrmIs with available structures (Fig. 2). Asp148 of *A. aeolicus* TrmI is conserved in *P. abyssi*, *T. maritima*, *M. tuberculosis*, and *Homo sapiens*. Lys153 in *T. thermophilus* TrmI is an exception. Lys150 of *A. aeolicus* TrmI is not conserved and does not interact with AdoMet. Glu155 of *T. thermophilus* TrmI and Tyr155 of *P. abyssi* TrmI participate in the AdoMet binding in distinct manners. By contrast, Ser175 of *H. sapiens* Trm61 (PDB ID 2B25) is 4.7 Å away from the N6 amino group, and does not interact with AdoMet. The TrmIs from *T. maritima* (PDB ID 1O54) and *M. tuberculosis* (PDB ID 1I9G) have Ser and Ala residues, respectively. Although the only available structure of *T. maritima* TrmI is the substrate-free form, the Ser residue is located too far away to interact with AdoMet. Tyr172 of *A. aeolicus* TrmI is conserved in *M. maritima* TrmI, and is replaced by aliphatic residues in *T. thermophilus* TrmI, *M. tuberculosis* TrmI, and *P. abyssi* TrmI, and by Thr175 in *H. sapiens* Trm61. The side chain of Thr175 is 5.3 Å away from the N6 amino group (PDB ID 2B25), and its hydroxyl group does not coordinate any water molecules.

Two other differences are the presence of T-stacking by Phe98 in *A. aeolicus* TrmI (Fig. 1d), and the additional hydrogen bond to the ribose moiety by His130, observed in *T. thermophilus* TrmI (Fig. 1e). The presence of Phe98 is unique to *A. aeolicus* TrmI (Fig. 2), whereas the His residue at the corresponding position of His130 in *T. thermophilus* TrmI is shared by the *M. tuberculosis* and *H. sapiens* TrmIs. The binding modes for the other part of AdoMet are quite similar. They involve the hydrogen bond between N1 of the adenine moiety to the main-chain amide nitrogen, the interaction between the two hydroxyl groups of the ribose moiety and the Glu side chain, and the binding to the amino and carboxyl groups of the methionine moiety. For the methionine moiety, the Asp165 that interacts with the amino group is conserved, and the conformations of the main-chain amide groups that interact with the carboxyl group are quite similar.

## Summary

We have determined the crystal structure of TrmI from the extremely thermophilic bacterium *A. aeolicus*, and examined the similarities and differences regarding the recognition of the methyl donor AdoMet by *A. aeolicus* TrmI and the *T. thermophilus* and *P. abyssi* TrmIs. The recognition of the N6 amino group of the adenine moiety was the most diverse feature. Three residues are located where their side chains can approach the N6 amino group. Our comparative structural analyses revealed the different strategies adopted by these thermophilic species to form hydrogen bonds by using acidic and hydrophilic side chains. It is intriguing that the universal substrate AdoMet has become recognized in distinct manners by the TrmIs catalyzing the tRNA m^1^A58 modification, during the course of evolution.

## Abbreviations

AdoMet: *S*-Adenosyl-l-methionine
AdoHcy: *S*-Adenosyl-l-homocysteine
m^1^A: *N* ^1^-Methyladenosine
m^1^G: *N* ^1^-Methylguanosine
m^1^I: *N* ^1^-Methylinosine
m^3^C: *N* ^3^-Methylcytidine
m^3^Ψ: *N* ^3^-Methylpseudouridine
m^3^U: *N* ^3^-Methyluridine
IPTG: Isopropyl-1-thio-β-d-galactopyranoside
tRNA: Transfer RNA
PDB: Protein Data Bank
RMSD: Root-mean-square-deviation
